# The case for the complete decriminalisation of abortion care in Nepal and beyond

**DOI:** 10.1016/j.lansea.2025.100616

**Published:** 2025-06-17

**Authors:** Sabrina Germain, Mara Malagodi, Roshani Regmi, Radhika Saxena, Shivani Shinde

**Affiliations:** a The City Law School, City St George's, University of London, London, UK; b University of Warwick School of Law, University of Warwick, Coventry, UK; c iProbono, London, UK

**Keywords:** Abortion, Complete decriminalisation, Nepal, Sexual and reproductive health rights, Access barriers

## Abstract

Our Viewpoint makes the case for the complete decriminalisation of abortion in Nepal (and beyond) as a key strategy to removing barriers to abortion care. The criminal framing of abortion—even if subject to exceptions—creates legal barriers to accessing abortion, which then compound socio-cultural and medical barriers. Nepal represents fertile ground for abortion law reform centred on complete decriminalisation due to its pioneering constitutional approach to sexual and reproductive health rights. However, even in Nepal's liberal context, the enduring partial criminalisation of abortion hinders abortion access, especially for historically marginalised groups and in remote areas. This Viewpoint recommends complete decriminalisation in Nepal and South and Southeast Asian countries facing similar socio-legal barriers to abortion access. Our position as legal experts echoes calls for the complete decriminalisation of abortion by national and international medical professional bodies, which we hope will inform regional strategies to improve access to safe abortions.

## Introduction

This Viewpoint examines the barriers to accessing abortion care that the criminalisation of abortion creates and makes the case for the *complete* decriminalisation of abortion in Nepal and beyond. Our position statement as legal experts echoes the repeated calls for the complete decriminalisation of abortion by medical professional bodies at both national^1^ and international level,^2^ the latest 2022 WHO Abortion Care Guidelines,^3^ and the recommendations of international human rights bodies.^4^ Significantly, every jurisdiction in Asia—aside from the People's Republic of China—treats abortion by default as a crime and allows some exceptions under which abortion can be performed. Worldwide, only four other jurisdictions have completely decriminalised abortion: Canada (1988),^5^ Northern Ireland (2019),^6^ New Zealand (2020),^7^ and Australia (2023).^8^ The criminal framing of abortion exposes both abortion seekers and providers to harsh criminal sanctions. Depending on the specific legal framing, abortion seekers and providers may be falling foul of the criminal law, for instance, when abortions are not performed within the gestational limits or under the exceptions prescribed by law, or by a government-approved provider.

The impact of the criminalisation of abortion—whether complete (prohibited under *any* circumstances) or partial (permitted under a set of *exceptional* circumstances prescribed by law)—is twofold. First, the default legal treatment of abortion as a crime creates legal barriers to abortion care. Criminalisation compounds the stigma associated with pregnancy termination, translates into sparser information about abortion care, reduces the availability of abortion drugs, decreases the number of medical professionals undertaking abortion training, and as such increases the likelihood of unsafe abortions.^9^ In fact, abortion criminalisation adversely affects patients' reproductive autonomy and safety by having a chilling effect on medical professionals as the threat of criminal sanctions may prevent them from operating in the patient's best interest.^10^ Thus, criminal restrictions on abortion have resulted in complications for 9 million women and the deaths of 22,800 women annually worldwide.^11^ Second, the complete or even partial criminalisation of abortion compounds other existing non-legal barriers to accessing abortion care. In fact, legal, cultural, socio-economic, geographical, medical, and institutional barriers to abortion care act as mutually reinforcing mechanisms of systemic exclusion, especially for historically marginalised groups, and criminalisation amplifies their combined negative impact on abortion access.^12^

The complete decriminalisation of abortion can improve access to safe abortion care by removing the legal barriers and the chilling effect that these barriers create for both abortion seekers and providers; however, legal reform cannot by itself guarantee increased access to abortion care even if it is an indispensable starting point.^13^

Crucially, decriminalisation does not equate to deregulation. Decriminalisation requires “the complete removal of abortion from criminal law” so that it is regulated like any other medical procedure under healthcare frameworks, not the Penal Code.^14^ Complete decriminalisation does not lead to higher abortion rates,^5^ and with adequate safeguards it does not increase neither forced nor sex-selective abortions. These safeguards include robust informed consent provisions, availability of high-quality counselling and adequate time to consider choices fully, robust in-clinic procedures to ensure the voluntariness of a woman's consent (e.g., clinics should see each woman with no escort present to discuss any pressures on her).^15^ Significantly, decriminalisation reduces the incidence of unsafe abortions because criminalisation does not ultimately prevent pregnant individuals from seeking a termination, but it restricts their ability to do so safely.^16^

This Viewpoint recommends key reforms in the area of abortion law and focuses primarily on the complete decriminalisation of abortion as a key strategy to removing barriers to abortion care. Although a small jurisdiction, Nepal is the ideal case-study to appreciate the damaging impact of abortion criminalisation notwithstanding the country's highly progressive constitutional framing of sexual and reproductive health rights (SRHR). Thus, this emblematic case-study illustrates the need for legal reform not just in Nepal, but also in neighbouring jurisdictions with less advanced SRHR regimes but similar abortion laws. Our recommendations for legal reform centre on the complete decriminalisation of abortion; we hope they will help inform regional strategies, provide some solutions to navigate access barriers, and help rethink the case for the complete decriminalisation of abortion in Asia. Our legal analysis is informed by an intersectional approach and by the qualitative insights gained from a consultation with key Nepali stakeholders in the field of sexual and reproductive health—legal experts, medical practitioners, representatives of civil society and intergovernmental organisations, policy makers, and advocacy and campaign groups.^17^ On this basis, we make the case for the *complete* decriminalisation of abortion in Nepal and beyond as the necessary but not sufficient condition for improving access to abortion care.^18^

## The case for abortion law reform in Nepal and beyond

Nepal is one of the few jurisdictions where the complete decriminalisation of abortion seems achievable due to its progressive legal framing of SRHR. Yet the harmful effects of the partial criminalisation of abortion persist and impact disproportionately marginalised groups.^19^ Thus, Nepal imparts crucial lessons for other jurisdictions in South and Southeast Asia.

Up until 2002, abortion in Nepal was almost entirely criminalised and nearly 20% of female inmates had been convicted of abortion-related offences.^20^ Legislative reforms in 2002 and 2006 created exceptions to the criminal ban with gestational limits permitting abortion. Since 2007 Nepal's constitution began recognising SRHR explicitly and enabled the Supreme Court of Nepal to render groundbreaking decisions on abortion.^21^ In 2008, the Court held that abortion was integral to SRHR.^22^ In a later judgement, the Supreme Court ordered the government to provide free abortion services.^23^ Crucially, the Court held that abortion equates to the right to self-determination for women since it disproportionately impacts them. It explicitly stated that a woman has full bodily autonomy and must have the final word in deciding whether to have sexual relations, when to give birth to a child, and how to use her body. The Court also observed that abortion should be *completely* decriminalised since Nepali law does not classify the foetus as human life. Therefore, the foetus cannot be granted more importance than the protection of the physical and mental health of the woman, and a forced pregnancy and a forced continuation of pregnancy constitute violence against women.^24^

Lawmakers, however, did not fully implement this judgement. The text of the newly promulgated 2015 constitution includes SRHR but does not mention abortion explicitly.^25^, ^26^, ^27^ Moreover, the new legislation passed to enforce SRHR does not comply with the Supreme Court's recommendation for the complete decriminalisation of abortion.^28^ Both the Penal Code 2017 (s.5) and Safe Motherhood and Reproductive Health Rights Act (SMRHRA) 2018 (s.15) create a set of conditions under which a pregnant person can seek a lawful abortion, but any abortion outside these exceptions remains a criminal offence (Table 1).

**Table 1:** Grounds for lawful abortion under SMRHRA 2018.

Legal provision	Gestational limit	Ground	On demand
Section 15(a)	12 weeks	With consent of pregnant person	Yes
Section 15(b)	28 weeks	With consent of pregnant person, and danger upon the life of the pregnant woman or her physical or mental health may deteriorate, or a disabled infant may be born	No, doctor's consent required
Section 15(c)	28 weeks	With consent of pregnant person, and rape or incest	No, subject to evidence of rape/incest
Section 15(d)	28 weeks	With consent of pregnant person, and if HIV or any other incurable disease	No, subject to medical evidence
Section 15(e)	28 weeks	With consent of pregnant person, and due to defects occurred in the foetus (gestation), or that there is such defect in the foetus of the womb that it cannot live even after the birth, that there is condition of disability in the foetus (gestation) due to genetic defect or any other cause	No, health worker's consent required

Even in Nepal, a jurisdiction that is constitutionally progressive on SRHR, partial criminalisation continues to represent a major barrier to abortion care, while compounding other non-legal barriers. Nepali laws persist in treating abortion by default as a crime beyond 12-week-gestation, even if subject to exceptions. Moreover, abortion beyond 28-week-gestation remains illegal under any circumstance, even if the life of the pregnant person is at risk or in case of foetal anomalies.^29^ Significantly, the need for late term procedures is much more frequent in Nepal than in high-income countries, and it is more prevalent among socially disadvantaged groups in remote areas.^30^ Thus, the complete decriminalisation of abortion would enable a more equitable access to abortion care across the country.

The similarities between the legal framing of abortion in Nepal and in South Asian and Southeast Asian jurisdictions, alongside the negative impact of criminalisation on safe abortion access, support the case for complete decriminalisation not just in Nepal but also in neighbouring countries. Nepal's legal framing of abortion—even if more liberal—is comparable to other jurisdictions in South and Southeast Asia (Table 2). These legal frameworks clearly reflect the colonial genealogy of the criminalisation of abortion and mirror the approach to partial decriminalisation developed under English law post-1967. This approach is highly medicalised, foetus-centric, and criminalised as both abortion seekers and providers risk being exposed to criminal sanctions. Thus, the common legal matrix of abortion laws, the similar socio-cultural attitudes towards SRHR, and the patterns of intersectional marginalisation across the region explain the comparable negative impact of these legal barriers on safe abortion access. These similarities support the case for the complete decriminalisation of abortion and the role of legal reform in removing these barriers not just in Nepal, but also in neighbouring Asian jurisdictions.

**Table 2:** Abortion laws in Southeast and South Asia.^31^

Jurisdiction	Grounds for abortion	Criminal penalties
Nepal	➔ Gestation limit: 28 weeks of gestation ➔ Grounds: ◆ foetal impairment ◆ rape ◆ incest ◆ mental health ◆ physical health	• Penalties for abortion seeker^32^ • Penalties for provider • Penalties for person who assists • Penalties for non-consensual abortion and or negligence
India^33^	➔ Gestational limit: 20 weeks with the endorsement of one doctor; 20–24 weeks with the endorsement of two doctors; beyond 24 weeks a State-level Medical Board determines permissibility in specific cases (significant foetal abnormalities) ➔ Grounds: ◆ physical health ◆ mental health ◆ rape and incest ◆ foetal impairment ◆ economic/social reasons	• Penalties for abortion seeker • Penalties for provider • Penalties for person who assists • Penalties for non-consensual abortion and or negligence
Bangladesh	➔ Gestational limit: 12 weeks from the last known menstruation ➔ Ground: ◆ only to save a woman's life	• Penalties for abortion seeker • Penalties for provider • Penalties for person who assists • Penalties for non-consensual abortion and or negligence
Malaysia	➔ Gestational limit: up to 22 weeks of gestation ➔ Grounds: ◆ physical health ◆ mental health ◆ threat to life	• Penalties for abortion seeker • Penalties for provider • Penalties for person who assists
Thailand	➔ Gestational limit: permissible between 12 and 20 weeks ➔ Grounds: ◆ physical health ◆ mental health ◆ rape ◆ foetal impairment	• Penalties for abortion seeker • Penalties for provider • Penalties for person who assists • Penalties for non-consensual abortion and or negligence

The complete decriminalisation of abortion in Nepal would likely have a positive impact on the movements to decriminalise abortion in similarly situated Asian countries. It has been estimated that 45% of all global induced abortions between 2010 and 14 were unsafe, that “more than half of these unsafe abortions occurred in Asia, most of them in south and central Asia”,^34^ and that restrictive laws were associated with higher rates of unsafe abortions.^35^

## Barriers to abortion care in Nepal

Nepal's legal framework creates legal barriers to abortion care, which intersect and reinforce cultural, socio-economic, geographical, institutional, and medical barriers to access.

### Legal barriers

Stakeholders identified Nepal's legal framing of abortion as the primary barrier to abortion care.

(a) The criminalisation of abortion, even partial, increases the stigmatisation of abortion care. Stigma also legitimises criminalisation making human-centred legal reform towards *complete* decriminalisation harder to achieve.^36^ Stakeholders discussed the path-dependent nature of the default legal treatment of abortion as a crime, even when lawful exceptions are present. Criminalisation entails structural incentives to preserve the existing legal framework and disincentives towards legal reform. These include the desire to preserve legal stability alongside fear of backlash and the political cost of spearheading controversial reforms.
(b) Termination of pregnancy beyond 28 weeks of gestation is unlawful,^29^ exposing abortion seekers and providers to criminal liability, even if the pregnant person's life is at risk.
(c) The inconsistency between the gestational limits for the same grounds under the Penal Code and SMRHRA 2018^32^ creates uncertainty, even if the later law is to prevail.
(d) The definition of abortion under SMRHRA 2018 fails to distinguish between “abortion” and “miscarriage” increasing the risk of abortion seekers and providers falling foul of the criminal law.

The second legal barrier is low levels of legal literacy among medical personnel (who may refuse abortion care for fear of incurring criminal sanctions or who may incur criminal sanctions if unaware of abortion law), and abortion seekers (who may not understand which circumstances entitle them to a lawful abortion and forgo help). Stakeholders noted low levels of legal literacy surrounding SRHR among legal practitioners and lawmakers. Due to social stigma, SRHR as an area of legal practice and expertise is confined to a small number of primarily female legal practitioners, while most of the legal profession remains poorly trained in this area of law. Stakeholders also noted that lawmakers overall display a poor understanding of the difference between partial and complete decriminalisation of abortion care, and their implications.^37^

The third legal barrier has been the piecemeal regulation of abortion drugs.^38^ Some pharmacies have been allegedly distributing drugs without prescriptions, despite over-the-counter medical abortion drugs being prohibited. Untrained pharmacists appear to have dispensed unsafe and ineffective drugs without facing consequences, and abortion seekers have taken unsafe and/or unlawful abortifacients. Those having used abortifacients improperly, might refrain from seeking medical help in case of complications fearing criminal sanctions for unlawful abortion.^39^

### Cultural barriers

Stakeholders have identified in Nepal significant cultural barriers such as the deep stigma associated with abortion and unwanted pregnancies, often compelling abortion seekers to turn to unsafe methods.

Patriarchal norms in Nepali society restrict women's autonomy over reproductive choices. Intersectional inequalities, where gender intersects with other identity categories (e.g., caste, religion, ethnicity, marital status, sexuality etc.), further complicate access to abortion care.^40^ The complete decriminalisation of abortion could help garner attention and strategic investments in education and outreach programmes to raise awareness and reduce stigma around abortion for women, girls, and their immediate families.^41^

### Socio-economic and geographical barriers

Stakeholders emphasised barriers arising from Nepal's unique topography, particularly the stark differences between the care received and available in urban and rural/remote areas. Abortion seekers in rural areas face heightened challenges accessing abortion services, stigma from their communities, and lack of awareness of available resources.^19^ Rural healthcare infrastructures are subpar, compelling some abortion seekers to seek care from untrained individuals or to incur substantial costs travelling to cities for assistance. Inadequate transportation and lack of medical facilities hinder the delivery of timely and safe reproductive healthcare.

The legalisation of abortion significantly reduces maternal mortality linked to unsafe abortions, particularly in rural communities.^42^ In areas where female sex-selective abortion is prevalent, legalisation has also helped safeguard the rights of women, providing equitable access to medical procedures and reducing risks associated with illegal, unsafe abortions.^43^ Legal abortion services give women the autonomy to make reproductive decisions, thereby contributing to addressing gender imbalances and mitigating the socio-cultural pressures sometimes seen in rural areas.^44^

Recently, climate change has also aggravated Nepal's vulnerability to natural disasters.^45^ These calamities have destroyed the existing healthcare infrastructure, and their increased frequency has hindered the redevelopment of facilities, further limiting access to essential services. Interlocking inequalities exponentially increase barriers to abortion care for those most in need of state support making them most vulnerable to the criminal sanctions associated with unlawful abortions.^46^

### Medical access barriers

Stakeholders from the field of gynaecology and obstetrics pointed to three significant barriers to abortion access. First, a significant lack of integration of medical services potentially leading to unsafe abortions. The lack of coordination between healthcare providers and pharmacists has had women, particularly in rural areas, seek unauthorised abortion medication, often without prior medical consultations. Pharmacists also often lack knowledge on medical abortion medication, leading to incomplete abortions and related complications. The criminalisation of abortion then fulfils a chilling effect on the ability of patients with complications from unsafe/unlawful abortions to seek medical help, and on medical professionals to provide care given the risk that the criminalisation of abortions exposes them to. Decriminalisation is likely to lead these vulnerable groups to engage earlier with abortion care and to see a higher number of healthcare providers in rural areas dispensing safe abortions.

Second, continuing education to perfect knowledge around safe abortion techniques is severely lacking.^47^ Although safe abortion training programmes for nurses, medical officers, and consultants are available, they are sparse, leaving many medical practitioners without the opportunity to undertake these courses or attend refresher courses. Stakeholders from civil society highlighted that medical institutions lack the infrastructure and there were issues with data protection. Despite these concerns, the government has not devised policies to increase awareness, facilitate access to existing services, or develop a broader range of programmes supporting reproductive autonomy, perhaps because of the enduring criminal framing of abortion.

Effectively managing abortion complications at the district level will require the development of local medical resources and comprehensive training for providers. Human resource development can be achieved by training mid-level healthcare workers, including nurses and midwives, to handle safe abortion procedures and complications. Community-based support and referral systems are essential to ensure comprehensive care.^48^

Legal practitioners also identified issues with the governmental regulation of abortion services. Among them, the way in which the government allocates resources to abortion care, the low level of quality control of health services, the understaffing of public hospitals and health posts in remote regions, and the lack of financial incentives for health professionals to take up posts in remote areas. Stakeholders also lamented the absence of streamlined and transparent procedures regulating abortion care facilities, resulting in a lack of public awareness about the right to abortion and a difficulty in holding providers accountable.

Finally, the high volume of cases in tertiary centres, particularly in the Kathmandu region, highlighted issues with the health referral system. Nepal has a three-tier health system—local, primary, and tertiary centres—but due to ineffective referrals, even basic cases are being referred to tertiary centres, which should be only dealing with critical and complex cases. This adds to the tertiary centres' overwhelming patient load straining the entire health system and creating challenges around the delivery of high-quality abortion care. The poor actualisation of SRHR manifests itself also in the lack of extensive and grass-root medical training on abortion increasing the risk for abortion seekers to incur into delays or face a complete unavailability of abortion care. This thereby increases the risk of forced pregnancies or criminal sanctions for unlawful abortions.

## Recommendations for the decriminalisation of abortion in Nepal

We recommend legislative amendments and regulatory changes to improve access to abortion care in Nepal.

Legal reform:

• Abortion care must be removed from the criminal law framework, but sex-selective abortions must remain a criminal offence under Section 17 of SMRHRA 2018.
• The 28-week gestational time limit for abortion must be removed to ensure equitable access to safe abortion.
• The law on abortion care and related services must include a provision for a permanent telemedical pathway (online or telephone consultations with a medical professional), similar to the measures adopted during the COVID-19 pandemic.^49^
• The law on abortion must guarantee that costs associated abortion services including post-abortion care (e.g., transportation, sick leave, etc.) are covered by a government sponsored programme.

Corollary recommendations for the government:

• Order local and primary medical centres to provide abortion services to reduce patient loads and inadequate referrals to tertiary medical centres.
• Enhance professional medical and pharmaceutical regulations in the area of reproductive health, e.g., medical professionals should be required to undertake VCAT training, and the abortion drugs regulation should be revised (quality assessments and monitoring of drug use).
• Update national health management information systems to ensure accurate data collection for comprehensive monitoring of abortion care facilities, providers, and drug availability. This will help address institutional failings and assess the impact of proposed legal and regulatory changes. This update should coincide with a revision of Nepal's confidentiality and data protection laws.
• Develop a comprehensive sex-education curriculum for secondary schools to address cultural barriers.

## Contributors

SG conceptualisation, formal analysis, writing original draft, review and editing.

MM formal analysis, writing original draft, review and editing.

RR formal analysis writing original draft.

RS project administration, writing original draft.

SS writing original draft.

## Declaration of interests

We declare no competing interest.

## Appendix A Supplementary data

Appendix-Methods

Nepali Abstract
