# Supplementary material for: The case for the complete decriminalisation of abortion care in Nepal and beyond
Source: Lancet Reg Health Southeast Asia. 2025 Jun 17;38:100616. doi: 10.1016/j.lansea.2025.100616 (PMC12213255; doi:10.1016/j.lansea.2025.100616)
Supplement: Appendix-Methods [file mmc1.docx]

APPENDIX WITH DETAILED METHODS

**THE CASE FOR THE COMPLETE DECRIMINALISATION OF ABORTION CARE IN NEPAL AND BEYOND**

This Viewpoint article adopts a mixed methods approach to make the case for the complete decriminalisation of abortion in Nepal and beyond.

First, we have adopted a doctrinal approach to analyse the relevant legal material surrounding sexual and reproductive health rights (SRHR) in Nepal. These include constitutional provisions, statutes, and case law.

Second, we have adopted a comparative law method to analyse the SRHR framing in Nepal in comparison with neighbouring and cognate jurisdictions.

Third, we have undertaken a comprehensive literature review of the most recent policy guidelines and position statements by medical professional bodies at both national and international level, WHO, and international human rights bodies, alongside the latest relevant scholarship on abortion and SRHR law and policy.

Fourth, we have summarised the discussions we have had with key Nepali stakeholders in the field of sexual and reproductive health (legal experts, medical practitioners, representatives of civil society and intergovernmental organisations, policy makers, and advocacy and campaign groups) at a consultation we held in Kathmandu on 13 September 2024, on Barriers to Access to Abortion in Nepal.

With the participants’ permission, we summarised their private communications in this Viewpoint. The consultation process was not part of a research study. The private communications used in the Viewpoint article are the product of the semi-structured discussions during the consultation. Stakeholders were divided into 4 sector-specific groups consisting of lawyers, civil society representatives, medical professionals, and policy makers to discuss specific questions related to barriers to abortion access and develop policy recommendations, but these discussions did not take the form of a research interview.

This is the list stakeholders who took part in the consultation:

1. Dr Aruna Karki, Kathmandu Model Hospital;
2. Dr Shivani Rayamajhi, YoSHAN (Youth Led SRHR Advocacy Nepal);
3. Anjila Thapa, YoSHAN
4. Dr Niharika Khanal, YoSHAN
5. Pushpa Joshi, YoSHAN
6. Reena Lama, Development worker, Activist, FAITH (Friends Affected and Infected Together in Hand)
7. Shweta Karna, Advocacy and Campaign Coordinator, Beyond Beijing Committee (BBC) Nepal
8. Samikshya Pant, Intern, People Forum for Human Rights
9. Anisha Buda, National Indigenous Women Forum (NIWF)
10. Shreya Parajuli, Informal Service Sector Centre (INSEC)
11. Anurag Subedi, Paramount Legal Advisory Services Pvt. Ltd.
12. Samir Ghimire, UNFPA Nepal
13. Reshmi Sunar, UNFPA Nepal
14. Renu Ghimire, National Judicial Academy, Nepal
15. Sunil Babu Pant, first openly gay Constituent Assembly Member of Nepal, Activist
16. Ranju Darshana, Politician, Mental Health Advocate, Bibeksheel Sajha Party
17. Dechen Lama, Satya Raksha Law Firm & Consultancy

Please find [HERE](https://doc-10-c4-apps-viewer.googleusercontent.com/viewer/secure/pdf/hbt4bu5bjiv1b5uf3ggcapdt8lujhjrn/3305m7ous5bdpobiil4235oi3rg9v3pq/1748344500000/drive/03317848860436072806/ACFrOgAWblwVabA7A2hbS1wG_JNNHa4KiHRBrjyCGU6b-OrXIngj0O80h8esAWFAhDw2AtZl8ziQgbT7yTmp_IzOWd6-7Ab2lV5kHPO2HJTM7m4IuFGdyHCet3odt3mHJ5KDoznwcD3tMHS9-aLfE2O_DVAoYV0NgvRupyEPffbFbxEdI6NhgJKBK5N2lCODQT_Mp4IVoRFcs9YqcIOB0Cglp6V6uip8P1ujvXD324BZP3cD8ImnNlNIKXt1yxA=?print=true&nonce=vando316e59ui&user=03317848860436072806&hash=3um6rm3dfdvre0720f9abfvd62ppss08) the forms with the permissions from the stakeholders who participated in the consultation.
