# Supplementary material for: The case for the complete decriminalisation of abortion care in Nepal and beyond
Source: Lancet Reg Health Southeast Asia. 2025 Jun 17;38:100616. doi: 10.1016/j.lansea.2025.100616 (PMC12213255; doi:10.1016/j.lansea.2025.100616)
Supplement: Nepali Abstract [file mmc2.pdf]

"नेपाल र अन्तर्राष्ट्रिय सन्दर्भमा गर्भपतन सेवाको पूर्ण गैर-अपराधीकरणको औचित्य: सारसंक्षेप।"

हाम्रो दृष्टिकोणले नेपाल (र अन्तर्राष्ट्रिय क्षेत्र) मा गर्भपतन सेवाको पूर्ण अपराधीकरणलाई गर्भपतन सेवामा पहुँचको अवरोध हटाउने प्रमुख रणनीतिको रूपमा प्रस्तुत गर्दछ। गर्भपतनको आपराधिक संरचना - अपवादको विषय भए पनि - गर्भपतनमा पहुँच गर्न कानुनी अवरोधहरू सिर्जना गर्दछ, जसले सामाजिक-सांस्कृतिक र चिकित्सा अवरोधहरूलाई जटिल बनाउँछ। यौन तथा प्रजनन स्वास्थ्य अधिकारको लागि नेपालको अग्रगामी संवैधानिक दृष्टिकोणका कारण पूर्ण अपराधीकरणमा केन्द्रित गर्भपतन कानुन सुधारको लागि नेपाल उर्वर भूमि हो। यद्यपि, नेपालको सन्दर्भमा पनि, गर्भपतनको निरन्तर आंशिक अपराधिकरणले गर्भपतनको पहुँचमा बाधा पुऱ्याउँछ, विशेष गरी ऐतिहासिक रूपमा सीमान्तकृत समूहहरू र दुर्गम क्षेत्रहरूमा। यो दृष्टिकोणले नेपाल र दक्षिण तथा दक्षिणपूर्वी एसियाली देशहरूमा गर्भपतन पहुँचमा समान सामाजिक-कानूनी अवरोधहरूको सामना गरिरहेका देशहरूमा पूर्ण -अपराधीकरण सिफारिस गर्दछ। कानूनी विशेषज्ञको रूपमा हाम्रो अडानले राष्ट्रिय र अन्तर्राष्ट्रिय चिकित्सा पेशेवर निकायहरूद्वारा गर्भपतनको पूर्ण अपराधीकरणको लागि आह्वानलाई प्रतिध्वनित गर्दछ, जसले सुरक्षित गर्भपतनमा पहुँच सुधार गर्न क्षेत्रीय रणनीतिहरूलाई सूचित गर्ने आशा लिएका छौँ।
